# CD38 Expression in a Subset of Memory T Cells Is Independent of Cell Cycling as a Correlate of HIV Disease Progression

**DOI:** 10.1155/2016/9510756

**Published:** 2016-03-14

**Authors:** Daniela Würsch, Christopher E. Ormsby, Dámaris P. Romero-Rodríguez, Gustavo Olvera-García, Joaquín Zúñiga, Wei Jiang, Santiago Pérez-Patrigeon, Enrique Espinosa

**Affiliations:** ^1^Department for Research in Immunology, National Institute for Respiratory Diseases, Calzada de Tlalpan 4502, 14080 Mexico City, DF, Mexico; ^2^Center for Research on Infectious Diseases (CIENI), National Institute for Respiratory Diseases, Calzada de Tlalpan 4502, 14080 Mexico City, DF, Mexico; ^3^Flow Cytometry Core Facility, National Institute for Respiratory Diseases, Calzada de Tlalpan 4502, 14080 Mexico City, DF, Mexico; ^4^Department of Medicine and Department of Immunology and Microbiology, Medical University of South Carolina, 173 Ashley Avenue, The Basic Science Building, Room 208D, Charleston, SC 29425, USA; ^5^Department of Infectious Diseases, National Institute for Medical Sciences and Nutrition, Vasco de Quiroga 15, 14080 Mexico City, DF, Mexico

## Abstract

In order to determine if the expression of the activation marker CD38 can correlate with HIV disease progression independently of cycling, we performed a cluster-based multivariate correlation analysis of total circulating CD4^+^ T cell counts and viral loads with frequencies of CD38 and Ki67 expression on CD4^+^ lymphocytes from patients with untreated HIV infection, stratified in maturation subpopulations, and subpopulation subsets defined by the expression of CXCR5, CXCR3, and CCR4. The frequencies of the activated phenotypes %CD38^+^ Ki67^−^ and %CD38^+^ Ki67^+^ of the CXCR5^−^ CXCR3^−^ CCR4^+^ (“pre-Th2”) central memory (T_CM_) cell subset clustered together, comprising a significant negative correlate of total circulating CD4^+^ T cell counts and a positive correlate of viral load in multivariate analysis. Frequency of cycling-uncoupled CD38 expression in “pre-Th2” T_CM_ cells was a negative correlate of total circulating CD4^+^ T cell counts in univariate analysis, which was not the case of their %CD38^+^ Ki67^+^. CXCR5^+^ CXCR3^−^ CCR4^−  ^T_CM_ cells were underrepresented in patients, and their absolute counts correlated negatively with their %CD38^+^ Ki67^−^ but not with their % CD38^+^ Ki67^+^. Our results may imply that CD38 expression either reflects or participates in pathogenic mechanisms of HIV disease independently of cell cycling.

## 1. Introduction

T cell activation is a strong predictor of CD4^+^ T cell loss in HIV infection [1], particularly when assessed by the expression of CD38, which shows a remarkable value as a predictor of HIV disease progression in diverse settings [1–3]. T cell activation has accordingly been deemed a possible indirect mechanism of CD4^+^ T cell depletion in HIV disease [4, 5].

A number of studies on activation have also measured the expression of the nuclear and perinuclear protein Ki67 initially considered to indicate proliferation [6, 7] and later delimited as an indicator that cells are in cycle [8] and undergoing turnover [9]. However, the expression of Ki67 does not always correlate with that of CD38, and these molecules show different predictive value depending on the T cell subset on which they are analyzed [5, 10–12]. In several studies Ki67^+^ CD4^+^ T cells comprise only a fraction of CD38^+^ cells [13–16], and these molecules show different expression dynamics during antiretroviral treatment and in other settings [17–19]. Therefore, it is important to investigate their relative contributions to the association of T cell activation and overall CD4 T cell loss.

The differences between CD38 and Ki67 as predictors may also reflect that their relationship with CD4^+^ T cell loss depends on the cell population that is studied which makes it potentially relevant to detect activation in different maturation subpopulations and additionally in relevant subsets within maturation subpopulations. Among subpopulations, central memory CD4 T cells (T_CM_ cells) have important self-renewal and differentiation capacities [20–22] and are crucial to the relative homeostasis of memory cells during the chronic phase of HIV infection [23–26].

Different subsets of T_CM_ cells have been identified by their expression of CXCR5, CXCR3, and CCR4 chemokine receptors. These subsets display specialized responses* in vitro* to TCR engagement or homeostatic cytokines, either proliferating and self-renewing (CXCR5^+^ CXCR3^−^ CCR4^−^  T_CM_ cells) or proliferating and differentiating to Th1 cells (CXCR5^−^ CXCR3^+^ CCR4^−^ “pre-Th1” cells) or to Th2 cells (CXCR5^−^ CXCR3^−^ CCR4^+^ “pre-Th2” cells) [20, 27]. This further subdivision of T_CM_ cells may be useful to investigate differential associations of CD38 and Ki67 with HIV disease progression, since their specialized functions correspond to those required by T_CM_ cells for their regenerative capacity in untreated HIV infection. Additionally, these chemokine receptors are by themselves important in T cell function and in HIV disease pathogenesis. CXCR5 is expressed by T_CM_ cells with B cell-help capacity [28] and by follicular helper cells, which are important in HIV control [29]. T cells expressing CXCR3 and CCR5, HIV's coreceptor, home to inflammatory sites [30], where CD4 T cell turnover is high [31]. CCR4 confers T cells the capacity to home to lung mucosal tissues [32], also critical in HIV disease [33]. Thus, we considered the subdivision of T_CM_ (and T_EM_) cells according to the expression of these receptors as potentially informative.

Our objective was to study both the joint or independent participation of CD38 expression and cell cycling (assessed by Ki67 expression), measured in different subsets within the different maturation subpopulations of circulating CD4^+^ and CD8^+^ T cells, as correlates of HIV disease progression, and to determine if they are mutually dependent.

## 2. Methods

This study was approved by the Institutional Boards of Instituto Nacional de Enfermedades Respiratorias Ismael Cosío Villegas (INER) and Instituto Nacional de Ciencias Médicas y Nutrición Salvador Zubirán (INCMN), Mexico. Blood samples were collected from 11 HIV^+^ antiretroviral-naive patients from the Department of Infectious Diseases of INCMN and from 11 healthy HIV^−^ controls. Both groups had 9 men and 2 women. Patients signed informed consent according to the Helsinki Protocol. Patients did not have any active opportunistic infection or malignancy, and none was receiving immunomodulatory drugs. CD4^+^ T cell counts were not available for one patient.

### 2.1. Phenotyping of Subsets of CD4^+^ and CD8^+^ Lymphocytes and Activation Phenotypes

**Figure 1:**
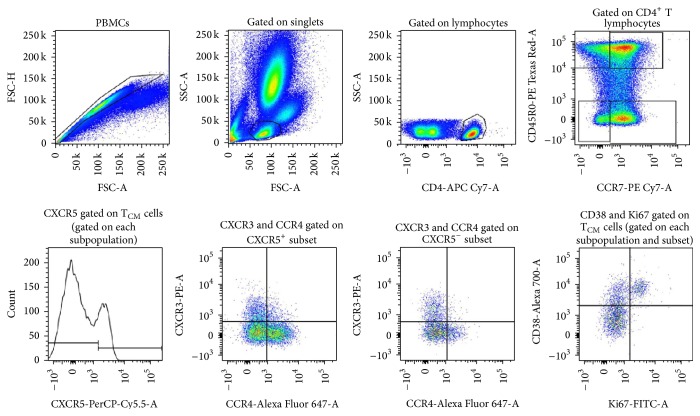
Successive analysis of CD4^+^ and CD8^+^ lymphocyte subsets and expression of CD38 and/or Ki67. The CD38 versus Ki67 plot, yielding three possible activation phenotypes, was analyzed in each gating stratum (layer); third plot, whole CD4 high lymphocytes (or CD8^+^ lymphocytes); each quadrant in the fourth plot, maturation subpopulations; each of eight possible patterns of expression of CXCR5 CCR4 and CXCR3 within each maturation subpopulation.

To determine frequency, activation phenotype, and functionality of maturation subsets of CD4^+^ T cells [20, 27], peripheral blood mononuclear cells (PBMCs) from HIV^+^ patients and healthy controls were obtained and processed completely immediately after sampling. Cells were incubated for 30 minutes at 4–8°C away from light with titrated biotin-conjugated monoclonal antibody specific for CD45RO (BioLegend, San Diego, CA, USA, Supplemental Material, Table  A available online at http://dx.doi.org/10.1155/2016/9510756), washed with phosphate-buffered solution containing 10% bovine serum albumin, and stained with Streptavidin conjugated with PE-Texas Red (BD Biosciences, San Jose, CA, USA, Supplemental Material, Table  A). This was followed by incubation with fluorochrome-conjugated monoclonal antibodies specific for surface molecules in the same conditions (Supplemental Material, Table  A). Cells were then washed with PBS (Lonza, Walkersville, MD, USA), fixed for 30 minutes with 500 *μ*L 4% p-formaldehyde (JK Baker, Mexico City, Mexico), and washed twice with 1 mL of a 1/2 dilution of Permeabilization Wash Buffer (10x BioLegend). Cells were then stained with anti Ki67-FITC (BD Biosciences, San Jose, CA, USA) in Perm/Wash buffer for 40 minutes on ice and away from light. Appropriate negative controls for each marker were used, consisting of cells stained with isotype controls plus the necessary fluorochrome-conjugated specific antibodies to eliminated spillover (Supplemental Material, Table  B). Cells were analyzed in a FACSCanto II cytometer (Becton Dickinson, San Jose, CA, USA) and analyzed with FlowJo software (Tree Star, San Carlos, CA, USA). Lymphocytes were identified by their side scatter and forward scatter properties, and, among them, we selected CD4^high^ cells (excluding all CD4^dim^ events) as well as CD8^high^ cells. Central memory cells (T_CM_) were delineated as CD45RO^high^ CCR7^+^  CD4^high^ or CD8^high^ lymphocytes. Effector memory cells (T_EM_) were CD45RO^high^ CCR7^−^ CD4^+^  CD4^high^  CD8^high^ lymphocytes, naive cells (T_N_) were CD45RO^−^ CCR7^+^  CD4^high^  CD8^high^ lymphocytes, and terminally differentiated cells were identified by the phenotype CD45RO^−^ CCR7^−^. This broadly used strategy excludes most of the possible contaminating cells other than CD4^+^ and CD8^+^ T cells (Figure 1).

### 2.2. Data Analysis

**Figure 2:**
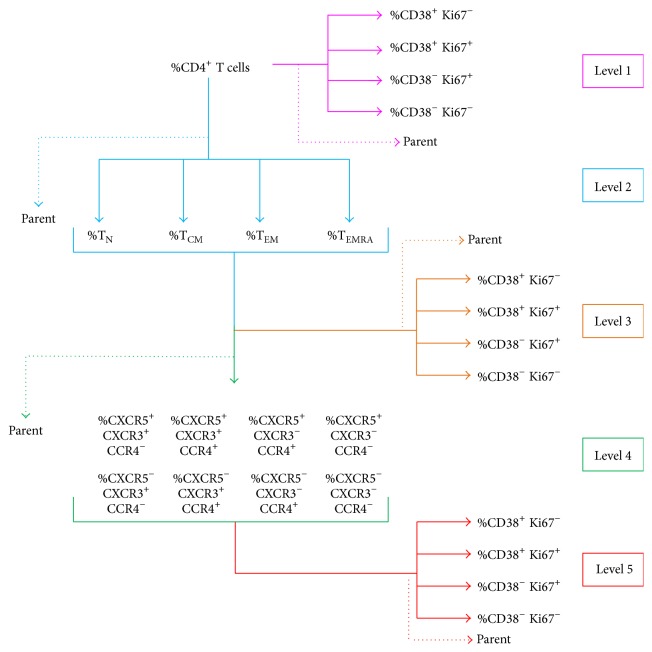
Groups of variables used in each cluster-based multivariate analysis. Subsequent gating generated groups of variables depicted as levels: level 1, CD4^+^ T cells; level 2, maturation subpopulations (naive, central memory, effector memory, and terminally differentiated); level 4, the eight possible combinations of expressions of CXCR5, CCR4, and CXCR3 within each maturation subset; and level 5, frequency of activation phenotypes within each subset. The frequencies (%) of activation phenotypes were analyzed at all higher levels. In each analysis level, the parent population was included to correct for differences in the percentage of the parent population as a confounder.

We obtained groups of variables for analysis by subsequent subgating, depicted as levels in Figure 2. We determined the frequency (%) of cells in each classification level of CD4^+^ T cells as follows (Figure 2):

 Level 1, activation phenotypes (CD38 and Ki67 expression patterns) on total CD4^+^ and CD8^+^ T cells.
 Level 2, maturation subpopulations on total CD4^+^ and CD8^+^ T cells.
 Level 3, activation phenotypes on subpopulations of level 2.
 Level 4, subsets within subpopulations, discriminated by the expression of chemokine receptors CXCR5, CXCR3, and CCR4 [20, 27].
 Level 5, activation phenotypes of each subset.

Gating was performed only on subpopulations comprising at least 300 events, which would assure meaningful percentages in the daughter population under the assumption of independent and identically distributed (iid) samples under a binomial distribution and considering only the last gate's variance. In this case, percentages derived from ≥300 events will have an 80% power for effect sizes of *h* ≥ 0.16.

Mann-Whitney test was used to analyze univariate differences between HIV^+^ patients and controls. Correlations were carried out by Spearman's ranked correlation. These tests were performed with StatView (Brain Power Inc., Calabasas, CA, USA) and Prism (GraphPad Software Inc., La Jolla, CA, USA) software.

### 2.3. Multivariate Analysis

Our analysis strategy started with a principal component analysis (PCA) to account for correlations between variables. PCA reduced variables to clusters of variables and generated a new variable consisting of the mean of their standardized values. Variables resulting from PCA were then utilized in multivariate analysis. We selected the PCA dimensions that cumulatively explained 80% of the dataset variance. As centroids for a *k*-means clustering algorithm we used for each variable the value of the individual that most contributed to the dimension to which the variable in question belonged. The final clusters had a 95% confidence measured by bootstrapping 1000 PCA clustering algorithms. This analysis was performed using R 2.1.1 with FactoMineR package [34]. Each cluster of variables had within it variables that had positive and negative estimates, and these are reported only as positive or negative, but the whole score for the dimension comprising all the variables was analyzed through normal multivariate logistic (in the case of HIV versus control) or linear (in the case of T CD4^+^ cell/mL of blood count or viral load) regressions, with associated *P* values.

### 2.4. Correction for Confounders

Statistical analysis required assuring that effects at a given subset were not influenced by differences in the parental population from which the subset was subgated. To control for these confounders, we included in the analysis of each cell group the frequency of its parent population (indicated in Figure 2 by a dotted arrow). For example, in the analysis using percentage of each activation phenotype in TN cells (level 3), the frequency of TN cells within CD4 T cells was also included.

## 3. Results

Patients had a median age of 33.1 years (range: 19 to 50), not differing significantly from controls (median: 27.3 years; range: 20 to 43). Patients had median 116 565 HIV RNA copies/mL blood (range: 1527–421 290), and median 323 CD4^+^ T cells/mm^3^ blood (range: 96–561).

### 3.1. Multivariate Correlates of Disease Progression

Cluster-based multivariate analysis showed a significant correlation of CD4^+^ T cell counts and viral load with one cluster composed of the %CD38^+^ Ki67^−^ of CXCR5^−^ CXCR3^−^ CCR4^+^  T_CM_ cells and the %Ki67^+^ CD38^+^ of the same subset as positive coefficients and the other phenotypes (Ki67^+^ CD38^−^ and Ki67^−^ CD38^−^) as negative coefficients (*P* = 0.036 for CD4^+^ T cell count, *P* = 0.030 for viral load, Table 1). This was the only multivariate correlate of CD4^+^ T cell counts that we found within all levels. Multivariate logistic regression showed a correlation of infection with a cluster containing %CD38^+^ Ki67^−^ of CXCR5^−^ CXCR3^−^ CCR4^+^  T_EM_ cells as the only positive coefficient with CD38 expression thus determining the significance (Table 1).

### 3.2. Linear Correlation of Relevant Activation Phenotypes with CD4^+^ T Cell Count

Since %CD38^+^ Ki67^−^ and %CD38^+^ Ki67^+^ of CXCR5^−^ CXCR3^−^ CCR4^+^  T_CM_ cells clustered as multivariate correlates of CD4^+^ T cell counts, we asked if both activation phenotypes were overrepresented among CXCR5^−^ CXCR3^−^ CCR4^+^  T_CM_ and T_EM_ cells from patients and if each one separately correlated with CD4^+^ T cell counts.

**Figure 3:**
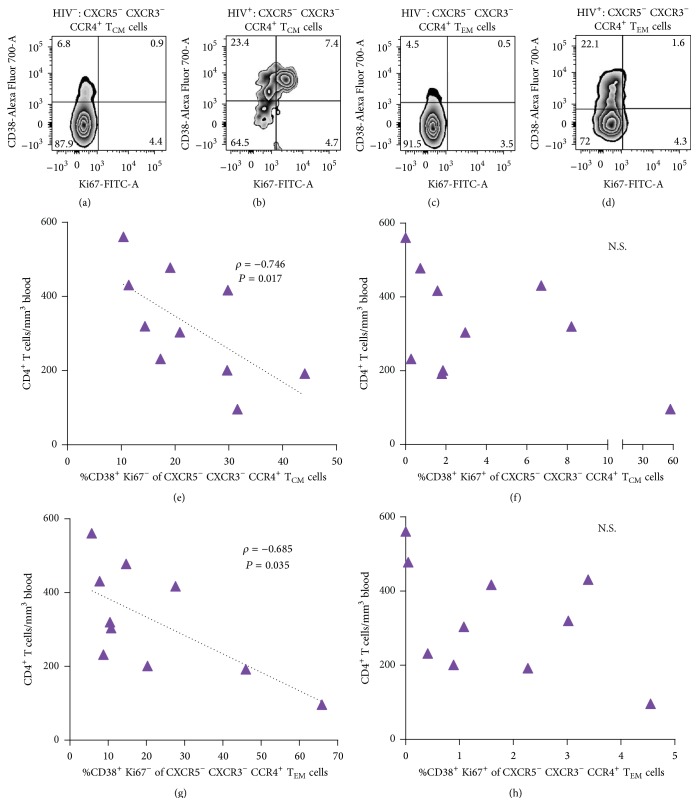
Relevant activation phenotypes and univariate correlation with CD4^+^ T cell counts. (a) Contour plot of CD38 and/or Ki67 expression in the CXCR5^−^ CCR4^+^ CXCR3^−^ subset of T_CM_ cells from a representative HIV^−^ control and a representative HIV^+^ patient. (b) CD38 and/or Ki67 expression on the CXCR5^−^ CCR4^+^ CXCR3^−^ subset of T_EM_ cells from a representative HIV^−^ control and a representative HIV^+^ patient. Numbers in each quadrant correspond to the group's mean frequency, as a percentage of the CXCR5^−^ CXCR3^−^ CCR4^+^ subset. (c) Correlation of total circulating CD4^+^ T cell counts in HIV^+^ patients with the percentage of CXCR5^−^ CXCR3^−^ CCR4^+^  T_CM_ cells with the CD38^+^ Ki67^−^ phenotype or (d) with the CD38^+^ Ki67^+^ phenotype. (e) Correlation of total circulating CD4^+^ T cell counts in HIV^+^ patients with the percentage of CXCR5^−^ CXCR3^−^ CCR4^+^  T_EM_ cells with the CD38^+^ Ki67^−^ phenotype or (f) with the CD38^+^ Ki67^+^ phenotype. Analysis was made with Spearman's correlation.

Among the three possible activation phenotypes (CD38^+^ Ki67^−^, CD38^+^ Ki67^+^, and CD38^−^ Ki67^+^), the CD38^+^ Ki67^+^ phenotype had an increased frequency in patients' CXCR5^−^ CXCR3^−^ CCR4^+^  T_CM_ cells, compared with their counterparts from controls (*P* = 0.009, Figures 3(a) and 3(b), upper right quadrants), and comprised the majority of Ki67^+^ cells, as previously described [35]. Contrastingly, a mean 23.4% of patients' CXCR5^−^ CXCR3^−^ CCR4^+^  T_CM_ cells showed Ki67-uncoupled CD38 expression (CD38^+^ Ki67^−^, Figure 3(b)), which comprised a majority of all CD38^+^ cells, and were also significantly more frequent, compared with controls (*P* = 0.0003).

Patients' T_EM_ cell subset with the same pattern of expression of chemokine receptors (CXCR5^−^ CXCR3^−^ CCR4^+^) showed an increased percent of Ki67-uncoupled CD38 expression (mean 22.1%, Figures 3(c) and 3(d), upper left quadrants), also significantly greater than its percentage among the controls' counterparts (*P* = 0.0001). Notably, the frequency of cells coexpressing CD38 and Ki67 did not differ between patients and controls and was negligible (Figures 3(c) and 3(d), lower right quadrant).

The frequency of the CD38^+^ Ki67^−^ phenotype on CXCR5^−^ CXCR3^−^ CCR4^+^  T_CM_ cells (Figure 3(e)) and on CXCR5^−^ CXCR3^−^ CCR4^+^  T_EM_ (Figure 3(g)) showed a significant negative correlation with CD4^+^ T cell counts (*ρ* = −0.746, *P* = 0.017, and *ρ* = −0.685, *P* = 0.035, resp.), while the frequencies of the CD38^+^ Ki67^+^ phenotype on these subsets showed no significant correlation with CD4^+^ T cell counts (Figures 3(f) and 3(h)).

Cycling of other cell subsets, like CXCR5^−^ CXCR3^−^ CCR4^+^  T_EM_ cells, as well as CXCR5^+^ CXCR3^+^ CCR4^−^  T_CM_ and T_EM_ cells, was also negatively correlated with CD4^+^ T cell counts; however, these associations were lost in multivariate analysis.

### 3.3. The CD38^+^ Ki67^−^ Phenotype Is the Only Negative Correlate of an Underrepresented T_CM_ Cell Subset

**Figure 4:**
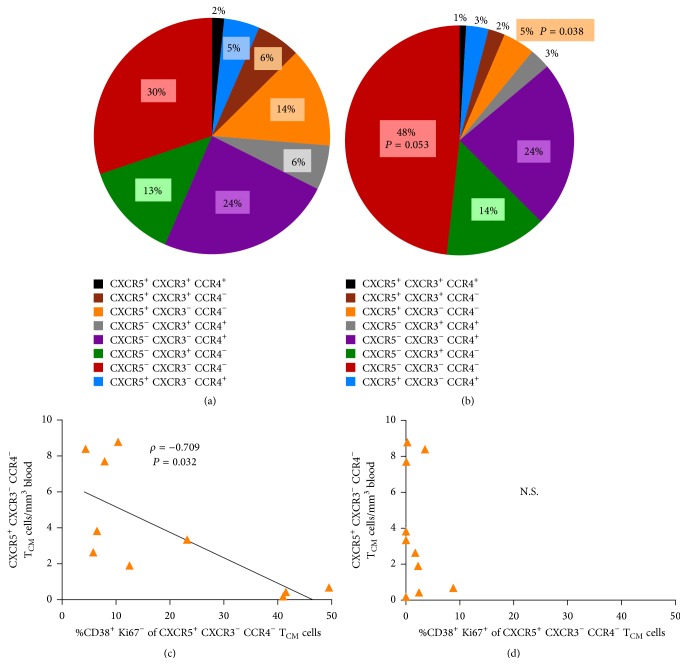
Effect of HIV on the relative subset composition of T_CM_ cells and its association with activation. Each slice of the pie chart corresponds to the mean percentage of each subset from a group, color-coded according to the list below (a) and (b). Pie charts correspond to controls' T_CM_ cells (a) and patients' T_CM_ cells (b). *P* values correspond to the comparison of patients and controls with Mann-Whitney's test. (c, d) Correlation of absolute counts of CXCR5^+^ CXCR3^−^ CCR4^−^  T_CM_ cells from patients with the percentage of this subset with the CD38^+^ Ki67^−^ activated phenotype (c) or with the CD38^+^ Ki67^+^ phenotype (d).

The proportion of CXCR5^+^ CXCR3^−^ CCR4^−^ cells among total T_CM_ cells (14 ± 4.165%, mean ± 1 SEM, Figure 4(a)) was significantly reduced in HIV^+^ patients (5%  ±  1.138%) compared with controls (*P* = 0.038, Figure 4(b)). The absolute counts of cells from this T_CM_ subset correlated negatively with their own %CD38^+^ Ki67^−^ (*ρ* = −0.709, *P* = 0.032) but not with their %CD38^+^ Ki67^+^ (Figures 4(c) and 4(d)), which constitutes another instance of the Ki67-uncoupled CD38 expression as a negative correlate of CD4 T cell counts.

There were analogous changes in the subset composition of T_EM_ cells, where both the underrepresentation of CXCR5^+^ CXCR3^−^ CCR4^−^ cells and the overrepresentation of CXCR5^−^ CXCR3^−^ CCR4^−^ cells were significant (*P* = 0.008, *P* = 0.014, correspondingly, not shown). T_N_ and T_EMRA_ cells were almost entirely CXCR5^−^ CXCR3^−^ and had only a small percentage of CCR4^+^ cells within CXCR5^−^ cells (not shown).

## 4. Discussion

In the present cross-sectional study of patients and controls, we found evidence that CD38 expression determines the correlation of activation and HIV disease progression independently of cell cycling, adjusted to a multivariate model that accounts for T cell maturation subpopulations and subsets within them. According to our analysis, cycling and noncycling cells from a CXCR5^−^ CXCR3^−^ CCR4^+^ subset of central memory CD4^+^ T cells (previously reported as “pre-Th2” cells [20]) clustered together on the basis of CD38 expression. In turn, this cluster was a negative correlate of circulating CD4^+^ T cell counts and a positive correlate of viral load. The small sample size of this study may have hampered the recognition of additional multivariate correlates of HIV disease progression. Also, since we did not determine additional indicators of activation, like, for instance, metabolic changes [36], the present study does not address the actual activation state of noncycling CD38^+^ cells, a sizable proportion of CD38^+^ cells. Despite these limitations, the thoroughness of our analysis evidences the fact that CD38 expression in a particular subset of CD4^+^ T cells has an inherent relevance as a correlate of CD4^+^ T cell counts, independent of whether CD38^+^ cells are in cycle.

Chronic T cell activation in HIV disease has been determined phenotypically by the detection of surface molecules (activation markers) like CD69, CD25 [37, 38], and notably CD38 and HLADR [2], which have different functions and are thus associated with different cellular processes [39]. The expression of Ki67, indicating cycling and turnover of T cells, has been used as a surrogate of activation markers [6]. It may correlate with the expression of phenotypic activation markers [35], and more importantly, it correlates with decreased CD4^+^ T cell numbers [5, 40]. Cycling may have a causal role in CD4^+^ T cell loss in HIV infection, since cycling CD4^+^ T cells from HIV^+^ patients show an increased turnover [9], are under cycle arrest [41], or die after entering S phase [42]. In contrast, whether activation markers, especially CD38 [2], reflect or participate in other processes leading to HIV disease progression remains to be elucidated.

The independence of CD38 in the present study as a correlate of disease progression has important implications. Either CD38 expression in “pre-Th2” T_CM_ cells is reflecting pathogenic processes leading to CD4 T cell loss or CD38 itself is participating in pathogenic mechanisms. In this regard, even though CD38 expression is highly correlated with T cell activation, its function in this process remains unknown. CD38 is a well-known ectoenzyme that catalyzes the transformation of NAD into ADPR, cADPR, and NAADP, and these “second messengers” can regulate the functionality of T cells [43–46]. Also regarding a possible role of CD38, we have previously reported that CD38^+^ CD4^+^  T_CM_ cells from HIV-infected patients show a response to TCR engagement dominated by IFN-*γ*, instead of IL-2, and disconnected of CD40L induction, as well as lack of response to CD28 costimulation [47, 48]. Such functionality could be less propitious to proliferation, a requirement for self-renewal and differentiation [21, 49], and might thus underlie the association of CD38 expression on “pre-Th2 T_CM_” (CXCR5^−^ CXCR3^−^ CCR4^+^) cells and overall CD4^+^ T cell loss in patients with HIV infection. These previous studies, along with our present findings, make a strong case for CD38 as an actor in pathogenesis of HIV disease. Mechanistic studies of this molecule in cells from patients with HIV are warranted.

There are additional ways in which activation of pre-Th2 T_CM_ cells could lead to depletion. Among human helper T cells, CCR4 is distinctly expressed by cells that express GATA3 and produce IL-4 when stimulated [50]. CCR4 expression in Th2-polarized central memory and effector memory cells directs homing to lungs [51–53] or other tissues with inflammation [54, 55], which are sites of increased CD4^+^ T cell turnover during chronic HIV infection [23, 31]. Importantly, CD38 expression may make them permissive to HIV infection [56, 57]. Additionally, IL-4 has been found to enable productive infection of CD38^+^ T cells by X4-tropic HIV-1 [58].

We observed a significant underrepresentation of CXCR5^+^ CXCR3^−^ CCR4^−^ cells among T_CM_ and T_EM_ cells from HIV^+^ patients. CXCR5^+^ CXCR3^−^ CCR4^−^  T_CM_ cells have been shown* in vitro* to self-renew by proliferating without differentiating after TCR-mediated or homeostatic cytokines [20]. Although the frequency of this T_CM_ subset was not a multivariate correlate of CD4^+^ T cell counts, it could be expected that T_CM_ cells from HIV-infected patients would be less able to maintain the memory pool, given their reduced percentage of CXCR5^+^ CXCR3^−^ CCR4^−^ cells. Additionally, the use of surface CXCR5 in our subset delineation brings forth the possibility that CXCR5^+^ CXCR3^−^ CCR4^−^  T_CM_ and T_EM_ cells could contain circulating cellular subsets with a follicular helper functionality [28, 59, 60]. Their relative underrepresentation contrasts with the previously reported expansion of dysfunctional follicular helper cells in lymph nodes from HIV^+^ patients [61]. The knowledge of the relationship between these two B cell-helping compartments could help understand this contrast.

## 5. Conclusions

Our study addresses the call for studying the biology of the diverse activation markers in the context of HIV infection [62]. Our findings indicate that focusing research on CD38's has informative potential, by possibly showing pathogenic mechanisms reflected by CD38 expression or mechanisms in which CD38 participates. Studying the subset level within the CD4^+^ maturation subpopulations is meaningful in HIV pathogenesis research.

## Supplementary Material

Table A shows the panel of fluorochrome-conjugated antibodies used for cell phenotyping by flow cytometry, including clone and origin. Table B shows the mixture of antibodies used to delineate positive and negative cells in the cytometry plots used to detect the expression of each marker.

## Figures and Tables

**Table 1:** Clusters of variables with significant correlation with HIV disease. *P* values correspond to a multivariate linear regression using main clusters as independent variables (see Section 2) and CD4^+^ T cell counts and viral load as dependent variable or status (HIV^+^ or HIV^−^) as dependent variable.

Correlate	Coefficient sign in cluster model	CD4^+^ T cells/mm^3^ blood^*∗*^		HIV RNA copies/mL blood	
Variables in cluster		Sign of regression coefficient	*P*	Sign of regression coefficient	*P*

T_CM_ CXCR5^−^ CCR4^+^ CXCR3^−^/Ki67^−^ CD38^+^	+	−	0.036	+	0.030
T_CM_ CXCR5^−^ CCR4^+^ CXCR3^−^/Ki67^+^ CD38^+^	+				
T_CM_ CXCR5^−^ CCR4^+^ CXCR3^−^/Ki67^+^ CD38^−^	−				
T_CM_ CXCR5^−^ CCR4^+^ CXCR3^−^/Ki67^−^ CD38^−^	−				

		HIV^+^ versus control			

T_EM_ CXCR5^−^ CCR4^+^ CXCR3^−^/Ki67^−^ CD38^+^	+	+	0.043		
T_EM_ CXCR5^−^ CCR4^+^ CXCR3^−^/Ki67^+^ CD38^+^	−				
T_EM_ CXCR5^−^ CCR4^+^ CXCR3^−^/Ki67^+^ CD38^−^	−				
T_EM_ CXCR5^−^ CCR4^+^ CXCR3^−^/Ki67^−^ CD38^−^	−				
