# Supplementary material for: CD38 Expression in a Subset of Memory T Cells Is Independent of Cell Cycling as a Correlate of HIV Disease Progression
Source: Dis Markers. 2016 Mar 14;2016:9510756. doi: 10.1155/2016/9510756 (PMC4808674; doi:10.1155/2016/9510756)
Supplement: Supplementary file 1 — Table A shows the panel of fluorochrome-conjugated antibodies used for cell phenotyping by flow cytometry, including clone and origin. Table B shows the mixture of antibodies used to delineate positive and negative cells in the cytometry plots used to detect the expression of each marker. [file 9510756.f1.pdf]

A. Antibody panel for phenotyping

Specificity	Fluorochrome	Detector	Clone	Volume	Isotype	Origin
CD4	APC-Cy7	780/60	RPA-T4	1μl	Mouse IgG1, κ	BD Biosciences
CD45RO	Biotin-Streptavidin PE Texas Red	610/20	UCHL1	0.5 μl /25 μl (1:1000)	Mouse IgG2a, κ	Biolegend/ BD Biosciences
CCR7	PE-Cy7	780/60	3D12	5 μl	Rat IgG2a, κ	BD Biosciences
CXCR3	PE	575/26	1C6/CXC R3	20 μl	Mouse IgG1, κ	BD Biosciences
CXCR5	PerCP-Cy5.5	695/40	J252D4	1.25 μl	Mouse IgG1, κ	Biolegend
CCR4	Alexa Fluor 647	660/20	1G1	1 μl	Mouse IgG1, κ	BD Biosciences
CD38	Alexa Fluor 700	730/45	HIT2	2 μl	Mouse IgG1, κ	Biolegend
Ki67	FITC	530/30	B56	20 μl	Mouse IgG1, k	BD Biosciences

**BD Biosciences, San Jose, California, USA. **Biolegend, San Diego, California, USA)*

B. Composition of the controls for the detection of each parameter

Antibody	Control for CCR7	Control for CXCR5	Control for CD45RO	Control for CXCR3	Control for CD38	Control for CCR4	control for Ki67
CD4-APC-CY7	✓	✓	✓	✓	✓	✓	✓
CD45RO-Biotin+ SAV-PE-Texas Red	✓	✓	Biotin mouse IgG2a-+SAV-PE-Texas Red	✓		✓	
CCR7-Pe Cy7	PE-CY7 rat IgG 2a	✓				✓	
CXCR5-PerCP-Cy5.5	✓	PerCP CY5.5-mouse IgG 2b			✓		
CCR4-Alexa Fluor 647		✓			✓	AF-647 mouse IgG 1	
CXCR3-Pe	✓	✓	✓	Pe-mouse IgG 1K			
CD38-Alexa Fluor 700					AF-700 mouse IgG1		
Ki67-FITC							FITC-mouse IgG1

"✓" indicates the inclusion of the specific antibody (left) in the control for the variable indicated as the column heading. Gray cells indicate that a specific antibody was not necessary in the control because compensation sufficed to eliminate completely the spill-over signal from a particular fluorochrome-conjugated antibody (left).
